# Facial asymmetry: a current review

**DOI:** 10.1590/2177-6709.20.6.110-125.sar

**Published:** 2015

**Authors:** Guilherme Thiesen, Bruno Frazão Gribel, Maria Perpétua Mota Freitas

**Affiliations:** 1 Professor, Universidade do Sul de Santa Catarina (UNISUL), Department of Orthodontics, Florianópolis, Santa Catarina, Brazil.; 2 Postdoctoral fellow in Orthodontics, University of Michigan, Ann Arbor, Michigan, USA.; 3 Adjunct professor, Universidade Luterana do Brasil (ULBRA), Canoas, Rio Grande do Sul, Brazil.

**Keywords:** Facial asymmetry, Orthodontics, Orthognathic surgery

## Abstract

The term "asymmetry" is used to make reference to dissimilarity between homologous elements, altering the balance between structures. Facial asymmetry is common in the overall population and is often presented subclinically. Nevertheless, on occasion, significant facial asymmetry results not only in functional, but also esthetic issues. Under these conditions, its etiology should be carefully investigated in order to achieve an adequate treatment plan. Facial asymmetry assessment comprises patient's first interview, extra- as well as intraoral clinical examination, and supplementary imaging examination. Subsequent asymmetry treatment depends on patient's age, the etiology of the condition and on the degree of disharmony, and might include from asymmetrical orthodontic mechanics to orthognathic surgery. Thus, the present study aims at addressing important aspects to be considered by the orthodontist reaching an accurate diagnosis and treatment plan of facial asymmetry, in addition to reporting treatment of some patients carriers of such challenging disharmony.

## Resumo

O termo assimetria é utilizado quando existe uma desigualdade entre as partes homólogas, afetando, assim, o equilíbrio entre as estruturas. A assimetria facial é comum na população, e muitas vezes se apresenta de forma subclínica. Entretanto, em alguns casos existe uma assimetria facial significativa que resulta tanto em problemas funcionais quanto estéticos. Nessas circunstâncias, a etiologia deve ser cuidadosamente investigada, para que seja possível elaborar um plano de tratamento adequado. A avaliação da assimetria facial é constituída por uma anamnese do paciente, exame clínico extra e intrabucal, além de exames complementares de imagem. O tratamento subsequente dessa assimetria depende da idade do paciente, etiologia e da magnitude da desarmonia, podendo envolver desde mecânicas ortodônticas assimétricas até a realização de cirurgia ortognática. Assim, o presente artigo busca abordar aspectos importantes para que o ortodontista possa realizar um adequado diagnóstico e plano de tratamento da assimetria facial, além de relatar o tratamento de alguns pacientes portadores dessa desafiadora desarmonia.

## INTRODUCTION

Many human body parts undergo development with bilateral symmetry. This implies that the right and left sides can be divided into identical mirror images. However, due to biological factors inherent to processes of development as well as environmental disturbances, perfect bilateral symmetry is rarely found.^1^

**Figure 1:**
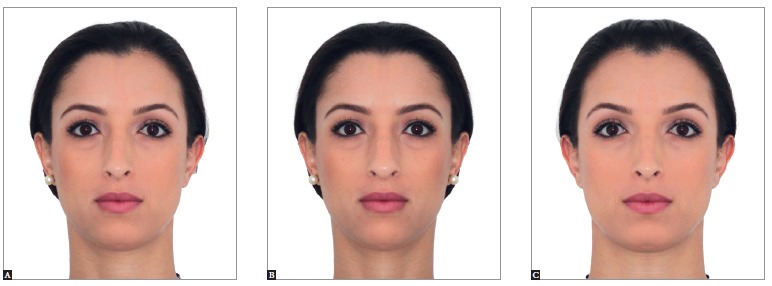
- Extraoral photograph in frontal view. Patient with relative symmetry (A) in which the median sagittal plane was determined and used as the basis to create mirror images on the right and left sides (B and C, respectively). Note that the original and mirror images differ from one another.

The face often presents with a mild degree of asymmetry. Nevertheless, slight asymmetry, also known as relative symmetry, subclinical asymmetry or normal asymmetry, ends up being unperceived by its carriers and everyone around them. It derives from the fact that the lower and midface develop from the medial and lateral nasal processes as well as maxillary and mandibular processes, and despite being intrinsically coordinated, these structures might imply failure of development or maturation of such embryonic processes.^2^^-^ 7 By editing the photographs of a pleasant face in frontal view, with its respective mirror image, three images are obtained: the original one, both right sides and both left sides. Assessment of these images evinces the existing bilateral discrepancies (Fig 1). 

However, whenever the degree of asymmetry is more severe, the condition is typically rendered noticeable, which negatively affects one's facial and smile esthetics.^5^^,^ 8 ^,^ 9

The orthodontic literature usually addresses changes in both anteroposterior and vertical directions; however, little attention is given to craniofacial changes in frontal view.^4^^,^ 8 Thus, the present study aimed at highlighting the major aspects of which the orthodontist should be aware regarding facial asymmetry, and their effects on orthodontic treatment of individuals affected by this condition, in addition to pointing out a few therapeutic options that could be applied to solve the issue.

## ETIOLOGY AND CLASSIFICATION

In the literature, a number of causal factors have been highlighted in the development of facial asymmetries. Chia et al^10^suggested that asymmetries could have pathological, traumatic, functional or developmental causal factors. Haraguchi et al^7^claimed that the etiology of facial asymmetry can be grouped into hereditary factors of prenatal origin and acquired factors of postnatal origin. 

Conversely, Cheong and Lo^2 ^reported that the causes of facial asymmetry can be grouped into three main categories: (I) congenital, of prenatal origin; (II) acquired, resulting from injury or disease; and (III) developmental, arising during development and of unknown etiology^2^ (Table 1). 

**Table 1:** - Major etiological factors of facial asymmetry, according to Cheong and Lo.

**Congenital factors**	**Acquired factors**	**Developmental factors**
Cleft lip and palate	Temporomandibular joint ankylosis	Unknown cause
Tessier clefts	Facial trauma	
Hemifacial microssomia	Children's radiotherapy	
Neurofibromatosis	Fibrous dysplasia	
Congenital muscular torticollis	Facial tumors	
Craniosynostoses	Unilateral condylar hyperplasia	
Vascular disorders	Parry-Romberg syndrome	
Others	Others	

Congenital changes associated with facial asymmetry comprise facial clefts, hemifacial microssomia, neurofibromatosis, anatomical changes at the base of the skull, congenital muscular torticollis, unilateral coronal craniosynostosis, positional plagiocephaly, among others. Acquired conditions causing facial asymmetry comprise trauma, fracture, arthritis and infection of the temporomandibular joint (TMJ), facial pathologies and tumors, hyperplasia or hypoplasia of the condyle, ankylosis of the temporomandibular joint, among others.^2^^,^ 3 ^,^ 11 ^,^ 12 ^,^ 13

In many cases, the etiology of facial asymmetry remains unknown and, for this reason, it is termed asymmetry of development. Such idiopathic asymmetries are common in the overall population, but are not found at an early age, appearing gradually throughout craniofacial development.^2^^,^ 14 The literature reports habitual mastication on one side, constant facial pressure during sleep exclusively on one side, deleterious oral habits or unilateral crossbite as being some of the causes of disharmony. The aforementioned factors would be responsible for increasing unilateral skeletal development. However, the hypotheses remain controversial and, due to lack of well-controlled longitudinal studies, cannot be scientifically validated.^7^^,^ 15 ^,^ 16 ^,^ 17

Lundstrom^15 ^also reported that facial asymmetries could be of genetic or non-genetic etiology, or a result of the interaction between the two.

As for the classification of craniofacial asymmetries, Bishara et al^18 ^assessed the structures involved and established that asymmetries could be classified as dental, skeletal, muscular or functional. 

With emphasis on mandibular changes, Obwegeser and Makek^19 ^suggested that asymmetries be classified as hemimandibular elongation or hemimandibular hyperplasia. Hemimandibular elongation might occur as a result of an increase of the condyle or the ramus in the vertical plane or an increase of the mandibular body in the horizontal plane. On the other hand, hemimandibular hyperplasia is characterized by an increase on one side of the mandible as a whole.

Hwang^20 ^developed a classification system for facial asymmetries, according to their main morphological features. The author established four types of asymmetry, based on skeletal analysis of deviation of the chin and bilateral difference between mandibular rami length. The four types of asymmetry would be as follows: patients with deviation of the chin and bilateral difference between mandibular rami length; patients with bilateral difference between mandibular rami length, only; patients with deviation of the chin, only; and patients with changes in volume on one side of the mandible, only, without deviation of the chin or discrepancy between mandibular rami length.

## PREVALENCE AND ASSOCIATED FACTORS

Epidemiological studies assessing facial asymmetries in orthodontic patients clinically found a prevalence ranging from 12% to 37% in the United States,^21^^-^ 23 23% in Belgium^24 ^and 21% in Hong Kong.^25^Whenever prevalence was assessed by radiographic examination, it presented values higher than 50%.^6^^,^ 11

In Brazil, Boeck et al^26 ^assessed the prevalence of skeletal deformities in a sample comprising 171 patients in need of orthodontic-surgical treatment. Their findings revealed a prevalence of 32% of asymmetries among the individuals assessed. Gribel et al^27^ assessed mandibular asymmetries by means of cone-beam computed tomography of 250 Class I subjects and found a prevalence of 44% of mild-to-severe asymmetries.

Severt and Proffit^22^ conducted a research with 1460 patients at the University of North Carolina and reported that 34% of individuals were found with a prevalence of facial asymmetry, with deviation of the chin being the most remarkable feature of asymmetry. Deviation of the chin was present in 74% of asymmetrical patients, with a frequency of lateral guidance of the upper and midface equal to 5% and 36%, respectively.

Therefore, deviation of the lower face is more frequent and greater in length than that of the upper and midface.^28^^,^ 29 A possible explanation would be the longer mandibular growth periods, in addition to the maxilla being rigidly attached to the stable region of synchondroses at the cranial base.^11^

Most studies on asymmetry claim that lateral guidance is most predominant on the left side of the face,^7^^,^ 27 ^,^ 30 with equal distribution among males and females.^6^^,^ 31 ^,^ 32 This occurrence could be explained by the dominant growth potential on the right side of the face, particularly considering the larger dimensions of the skull and the brain of individuals on the right side. Another potential innate mechanism causative of lateral guidance of the face might be related to the imbalanced development of neural crest cells. It has been speculated that neural crest cell migration happens earlier on the right side and tends to be delayed on the left side.^11^^,^ 33

As regards skeletal growth pattern, some authors claim that facial asymmetry is equally prevalent among skeletal Class I, II and II patients;^7^whereas other authors have shown that asymmetry is most frequently associated with Class III,^34 ^or less frequently associated with Class II.^22^In the vertical plane, facial asymmetry is apparently most prevalent among patients with a vertical growth pattern.^22^^,^ 34

## DIAGNOSTIC METHODS

In many patients, asymmetry results from a series of dentofacial changes and might lead to postural compensations that hinder the correct characterization of this disharmony. Thus, facial asymmetry must be assessed by thorough and judicious analysis conducted by means of a first interview, extra- and intraoral clinical examination, as well as supplementary diagnostic examination.^8^^-^ 10 ^,^ 35 ^,^ 36

During the first interview, patient's complaints and expectations should be assessed, and data on potential risks of infection, trauma or craniofacial pathologies collected.^37^

Clinical examination allows asymmetry to be assessed in sagittal, coronal and vertical dimensions, and it is the most important diagnostic tool in assessing the condition.^2^^,^ 18 Extraoral assessment comprehends visual inspection of facial morphology, associated with soft, hard tissues and TMJ palpation. A thorough facial analysis must be conducted, giving special attention to the center of the chin, leveling of lip commissures, and bilateral symmetry of gonial angles and mandibular body contours. At smiling, analysis should assess whether dental midlines coincide with facial midline, inclination of the occlusal plane and the amount of bilateral gingival exposure. Intraoral clinical examination should focus on assessing malocclusion, tipping of posterior and anterior teeth, crossbite and the presence of functional deviation of the mandible.^2^^,^ 20 ^,^ 37 ^,^ 38

In order to determine patient's facial midline, specific soft tissues landmarks and structures are used as reference. Thus, sagittal facial midline corresponds to a line perpendicular to the ground, passing through the glabella. Other landmarks of the upper and midface can also be used as reference, since these regions are less likely to present with bilateral asymmetry. Half the interpupillary distance, the subnasal point or the philtrum can also be used as reference to determine the midline in cases with some sort of imbalance near the glabella. Patient's tip of the nose and chin, however, present with greater variation.^8^^,^ 18

In order to have asymmetry assessed, patients must be in upright position, looking forward, with teeth in normal occlusion and relaxed lips. Additionally, having patient's upper and lower views often aids in determining asymmetry. A common procedure is the use of a piece of dental floss stretched from the region of the glabella to the lower chin, passing through the philtrum.^9 ^Another procedure used to assess inclination of the occlusal plane in vertical direction is asking the patient to bite a wooden sheet, so as to determine how the latter relates to the pupillary plane on both sides.^10^^,^ 37

According to Padwa et al,^39 ^an inclination of the occlusal plane higher than four degrees tend to cause remarkable asymmetry on patient's face. Special attention should be given to cases in which asymmetry is associated with progressive development of unilateral posterior open bite, since such fact might be a result of a pathology affecting the vertical dimension of the ramus or the mandibular condyle.^2^

In these patients, clinical examination should be supplemented with other diagnostic tools, such as casts, photographs, radiographs, tomography and bone scintigraphy, in order to locate and measure precisely the structures involved in asymmetry.^37^^,^ 40

Different methods of radiographic assessment are available to locate and measure the magnitude of facial asymmetry. Lateral cephalogram provides limited information, as structures on the right and left sides are overlapped. Additionally, magnification differs due to variation in the distance from the facial structures to the film and to the x-ray source. On the other hand, panoramic radiograph, frontal and submentovertex cephalograms might be considered useful tools. Skeletal as well as dental structures of the maxilla and mandible can be assessed and have right and left sides compared, thereby allowing potential bilateral differences to be evaluated. Nevertheless, those examinations present disadvantages, such as image magnification, overlapping structures and difficulty standardizing patient's head positioning, all of which hinder accurate assessment of facial asymmetry features.^27^^,^ 41 ^-^ 43

Thus, at present, the examination most often recommended to overcome the aforementioned disadvantages and allow thorough assessment of craniofacial asymmetries is computed tomography, especially cone-beam computed tomography (CBCT).^30^^,^ 44 ^,^ 45 Despite having a higher radiation dose when compared to a single conventional radiograph, a CBCT scan of the head usually produces an effective radiation dose that is lower than that of all supplementary radiographic examinations required for complete orthodontic records taken for asymmetry assessment purposes, further providing a more detailed diagnosis.^46^^,^ 47 The SedentexCT guidelines and the American Academy of Oral and Maxillofacial Radiology suggest the use of CT scans for assessment of facial asymmetries.^48^^,^ 49 It is also worth highlighting that CT scans allow tridimensional prototyped biomodels to be manufactured, which makes it easier for more complex surgical cases to be conducted.^37^^,^ 47

## ASSESSMENT OF STRUCTURES INVOLVED

Identifying the morphological features involved in the expression of facial asymmetry, in addition to patient's age and the magnitude of disharmony, is extremely important when coming up with an appropriate treatment plan. Thus, at the time of diagnosis, it is key to qualify and quantify all dental, skeletal, soft tissues and functional structures characterizing facial asymmetry.^10^^,^ 15

Asymmetry of dental origin alone does not usually lead to facial disharmony, but it might occasionally provide asymmetrical support to the tissues of the lip or affect smile harmony. In those cases, asymmetry might be caused by early loss of deciduous teeth, congenital single or multiple tooth loss, malposition of teeth, dental impaction, supernumerary teeth, among others.^18^

Skeletal asymmetry might involve a single basal bone, only; however, it usually affects the structures of the antagonist basal bone. Additionally, both the imbalanced and contralateral sides present with changes in structure. This is because whenever one side of bone development is affected, the opposite side is somehow influenced, which leads to growth compensation. In this context, the mandible is the structure most often associated with craniofacial asymmetries, with maxillary asymmetries often being secondary to asymmetrical mandibular growth. Mandibular asymmetries might involve the condyle, the ramus, the mandibular body and symphysis, all of which might undergo changes in size, volume or position. Therefore, determining which structures are involved, whether in the maxilla, mandible and/or another craniofacial region, in addition to establishing how much those structures have been affected, is essential to achieve a correct diagnosis.^2^^,^ 9 ^,^ 37

In general, skeletal deviation must be equal to or greater than 4 mm in order to render the asymmetry visible in an individual's face.^11^^,^ 36 ^,^ 50 ^-^ 52 Whenever the degree of asymmetry is lower, the condition tends to be considered mild and unperceivable. Nevertheless, asymmetry perception or blinding will also depend on individual characteristics, such as soft tissue thickness in that region. For this reason, other authors consider an asymmetrical face as having bone deviations equal to or greater than 2 mm.^6^^,^ 53 ^,^ 54

Masuoka et al^29 ^assessed the relationship between facial analysis and cephalometric indices by means of photographs in frontal view and posterior-anterior cephalograms of 100 asymmetrical patients. The authors concluded that whenever there is some discrepancy between skeletal measurements and subjective facial analysis, the influence of soft tissues structures should be considered key to characterizing asymmetry.

Importantly, facial asymmetry is usually presented with lower magnitude than skeletal asymmetry. According to the study conducted by Kim et al,^55 ^the degree of soft tissues asymmetry was lower than that of bone asymmetry in cases of deviation of the chin, inclination of the mandibular ramus in frontal view and inclination of the mandibular body also in frontal view. On the other hand, the degree of soft tissues asymmetry was greater than that of underlying hard tissues asymmetry, particularly regarding lip commissures angulation. Similarly, other studies^40^^,^ 50 ^,^ 56 reported that dental asymmetry is usually presented with lower magnitude than skeletal asymmetry, thereby compensating bone asymmetry.

## TREATMENT

Whenever coming up with an orthodontic or surgical treatment plan, great emphasis should be given not only to the diagnosis of asymmetry, but also to patient's final facial balance, as well as whether dental midlines coincide and proper occlusion has been achieved.^1^^,^ 8

Diagnosis of asymmetry can be easily achieved by the orthodontist working in cases involving significant deviation of dental midlines and absence of missing teeth, anomalies of shape or remarkable crowding on only one side of the arch.^8^^,^ 18 ^,^ 57 However, in other cases, facial asymmetry might be concealed by dental compensations, and if not properly diagnosed, it tends to be revealed throughout orthodontic treatment, thereby extending treatment time and hindering final outcomes. Once asymmetry has been diagnosed, the practitioner must wisely decide how to correct or treat it by means of compensations, bearing in mind potential limitations.^1^

Depending on patient's age and the severity of the condition, a variety of orthodontic and orthopedic options has been described in the literature with a view to correcting facial asymmetries. Of the many therapeutic approaches that have been reported, asymmetrical mechanics, asymmetrical extractions or surgical interventions are highlighted.^9^^,^ 37 For cases of mild asymmetry, asymmetrical mechanics and extractions tend to yield good results.^8^^,^ 10 ^,^ 58

**Figure 2:**
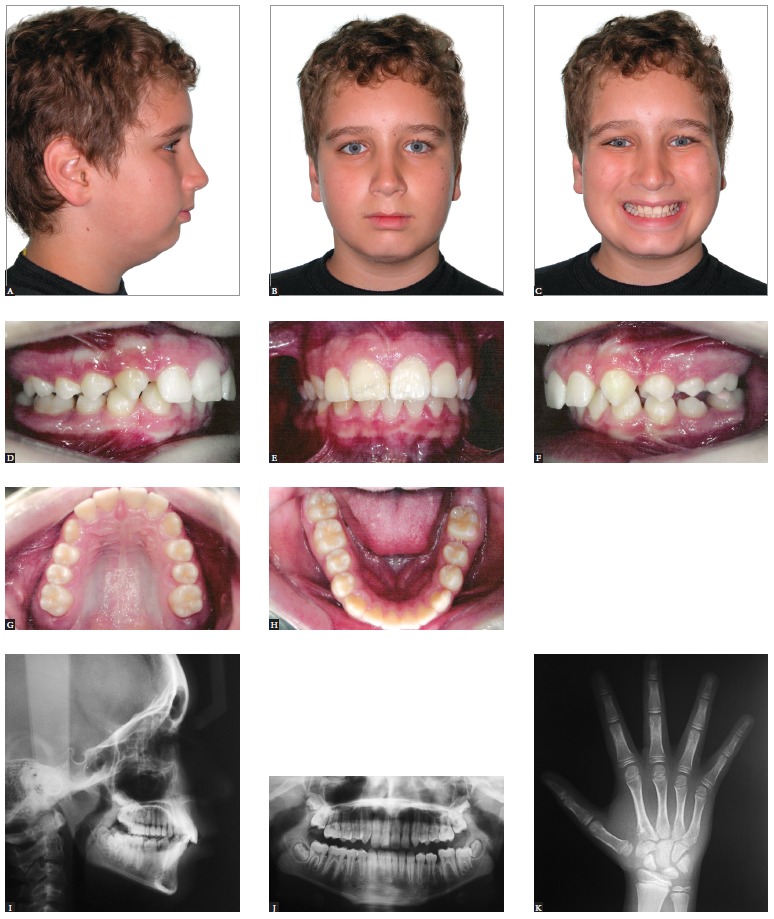
- Class II growing patient with mandibular deficiency. Presence of mild facial asymmetry with deviation of the chin to the left. Initial extraoral (A, B and C) and intraoral photographs (D, E, F, G and H), as well as profile, panoramic and carpal radiographs (I, J and K).

**Figure 3:**
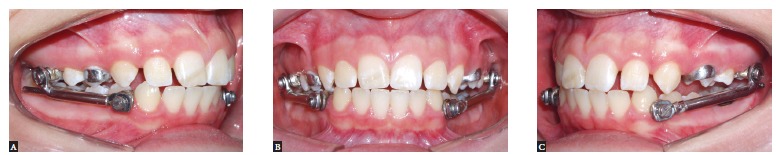
- Telescopic mechanism of the Herbst appliance in place. Asymmetrical mandibular advancement aiming at correcting skeletal occlusal and facial asymmetry. Lateral intraoral photographs on the right side (A), in frontal view (B) and on the left side (C).

**Figure 4:**
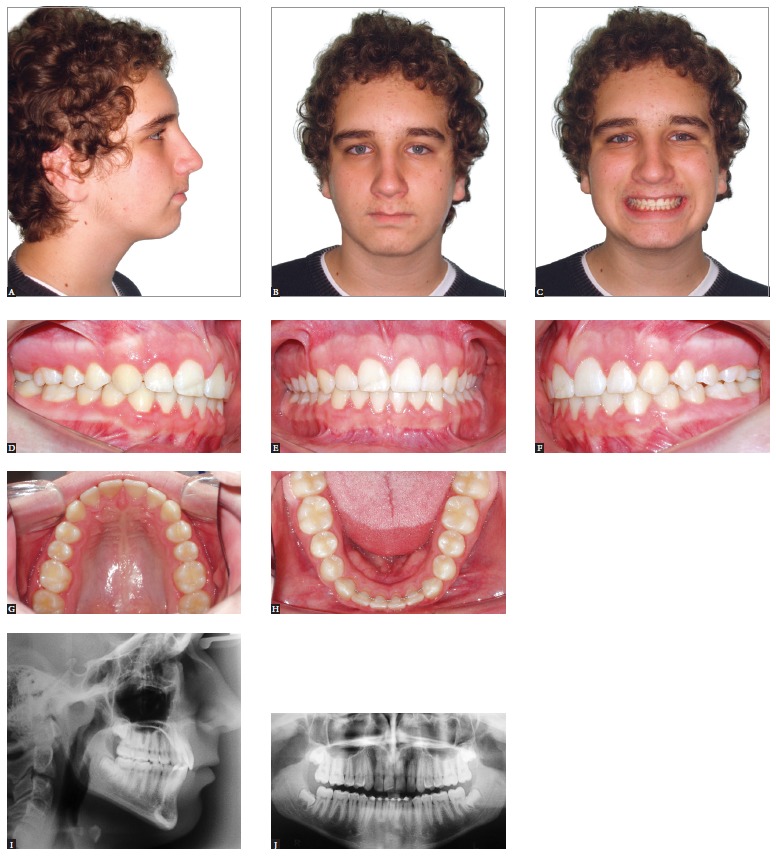
- Treatment outcomes for the patient presented in Figure 2, after the second phase of treatment conducted with full fixed orthodontic appliance. Final extraoral (A, B and C) and intraoral (D, E, F, G and H) photographs. Profile and panoramic radiographs (I and J).

As for growing patients, orthopedic asymmetrical approaches might be implemented (Figs 2 to 4). For adult patients in whom growth has ceased, asymmetrical mechanics has been recommended to solve disharmony by means of compensation. Achieving effective correction of asymmetry by means of asymmetrical activation of orthodontic and orthopedic appliances might be considered an effortful procedure; however, provided that basic biomechanical principals be followed, the use of asymmetrical resources becomes an ordinary and less intimidating procedure.^1^^,^ 9

**Figure 5:**
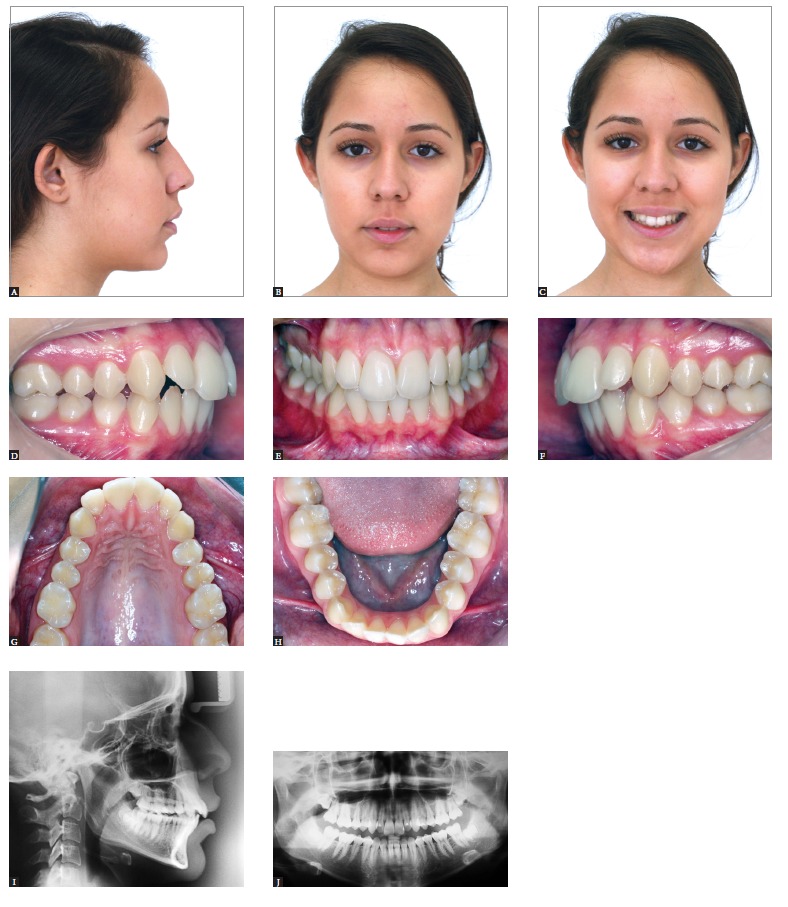
- Patient presenting Class II malocclusion on the right side, with negative cephalometric discrepancy and discreet crowding in both arches. Mild facial asymmetry with deviation of the mandible to the right. Initial extraoral (A, B and C) and intraoral photographs (D, E, F, G and H), as well as profile and panoramic radiographs (I and J).

**Figure 6:**
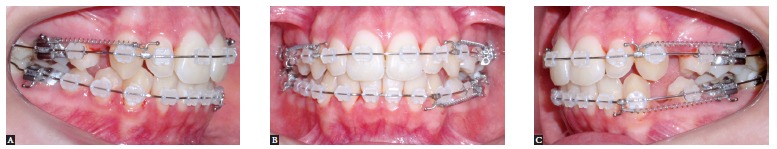
- Corrective orthodontic treatment with protocol including three extractions (teeth #14,24 and 34). Extraction in the mandibular arch was recommended for correction of lower dental midline coinciding with patient's median sagittal plane, in addition to correcting protrusion and crowding of mandibular anterior teeth. Extractions in the maxillary arch were carried out to correct protrusion, crowding and overjet. Lateral intraoral photographs on the right side (A), in frontal view (B) and on the left side (C).

**Figure 7:**
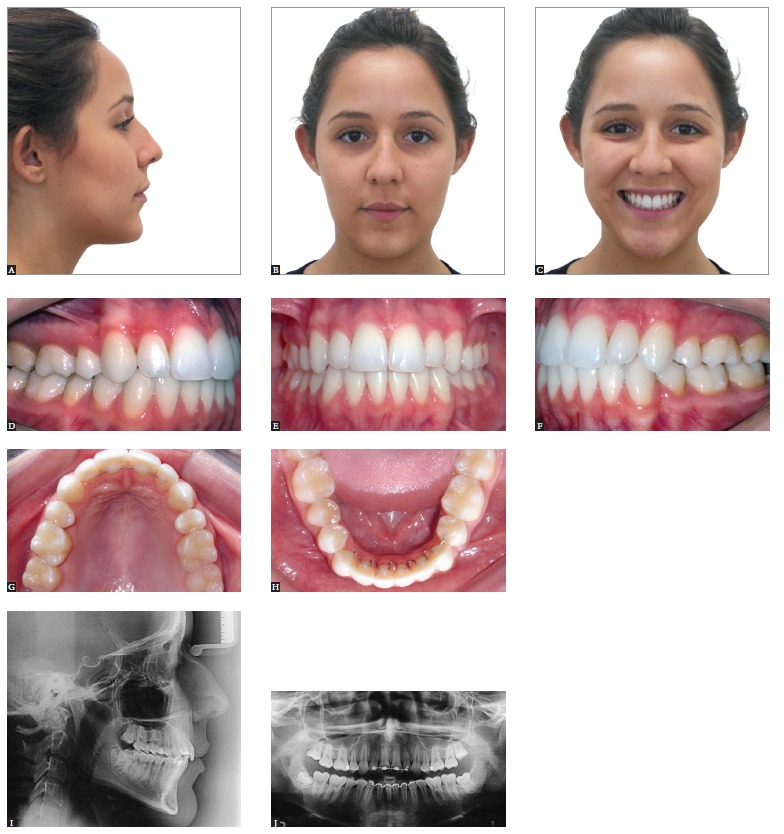
- Treatment outcomes for the patient presented in Figure 5. Final extraoral (A, B and C) and intraoral (D, E, F, G and H) photographs. Profile and panoramic radiographs (I and J).

Asymmetrical extractions, on the other hand, are a means of gaining the space required to correct potential discrepancies such as crowding and incisors proclination, in addition to compensating existing facial asymmetry (Figs 5 to 7). Anchorage control needs to be carefully analyzed, so that specific teeth are extracted with a view to allowing dental movement and thus correction of asymmetry. Therefore, more severe cases presenting significant asymmetrical occlusion can be corrected by means of routine orthodontic techniques.^9^

**Figure 8:**
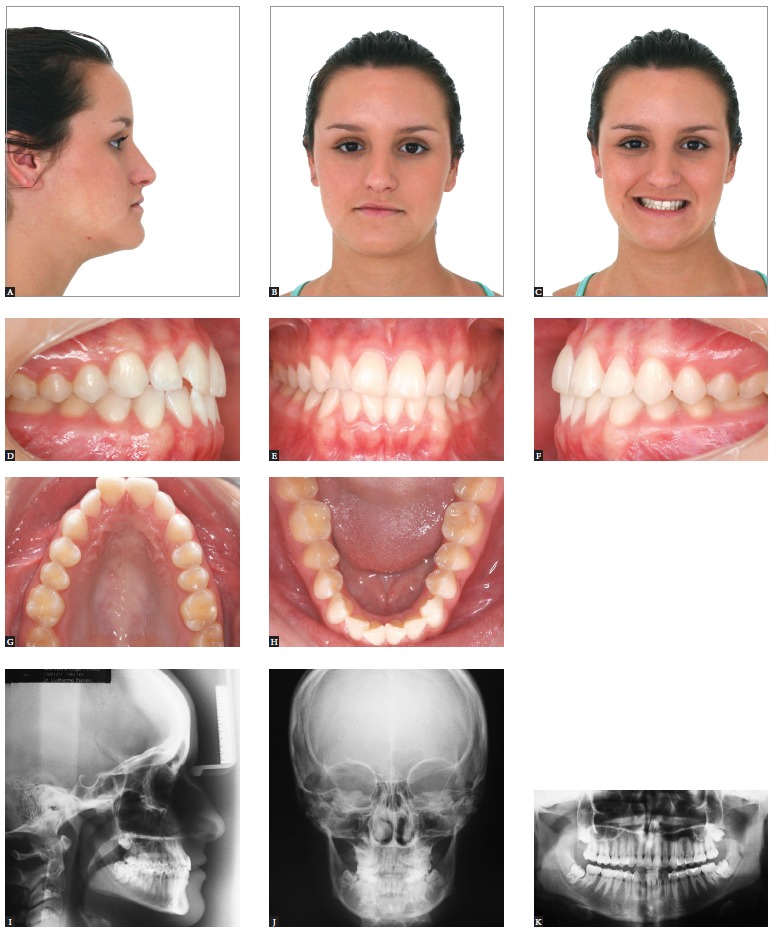
- Class I mature patient with asymmetry evinced by lateral deviation of the chin, in addition to vertical difference in leveling between lip commissures and inclination of the occlusal plane in frontal view. Initial extraoral (A, B and C) and intraoral photographs (D, E, F, G and H), as well as profile, posterior-anterior and panoramic radiographs (I, J and K).

In cases of severe facial asymmetry (Figs 8 to11), the treatment of choice should be a combination of Orthodontics and orthognathic surgery. Depending on the degree of dental, skeletal or soft tissue asymmetry, orthodontic treatment or surgical movement must be carried out asymmetrically, so as to achieve symmetry by the end of the therapy.^14^^,^ 37 Ideally, in those cases, orthodontic mechanics must be employed with a view to correcting potential dental compensations in the three planes of space. Special attention should be given to torque of posterior teeth, as it usually differs on the right and left sides in a physiological attempt to compensate lateral skeletal disharmony by causing dental changes.^9^

**Figure 9:**
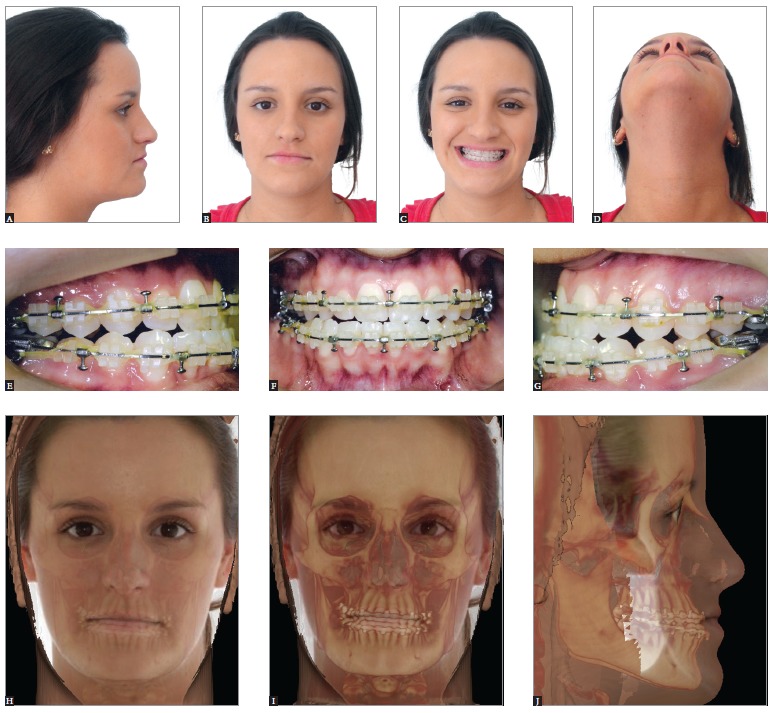
- Clinical aspect after presurgical orthodontic preparation carried out with a view to correcting dental tipping at their basal bones. The three planes of space must be considered. Extraoral (A, B, C and D) and intraoral photographs (E, F and G), as well as CT scans with soft tissues overlapping hard tissues (H, I and J).

**Figure 10:**
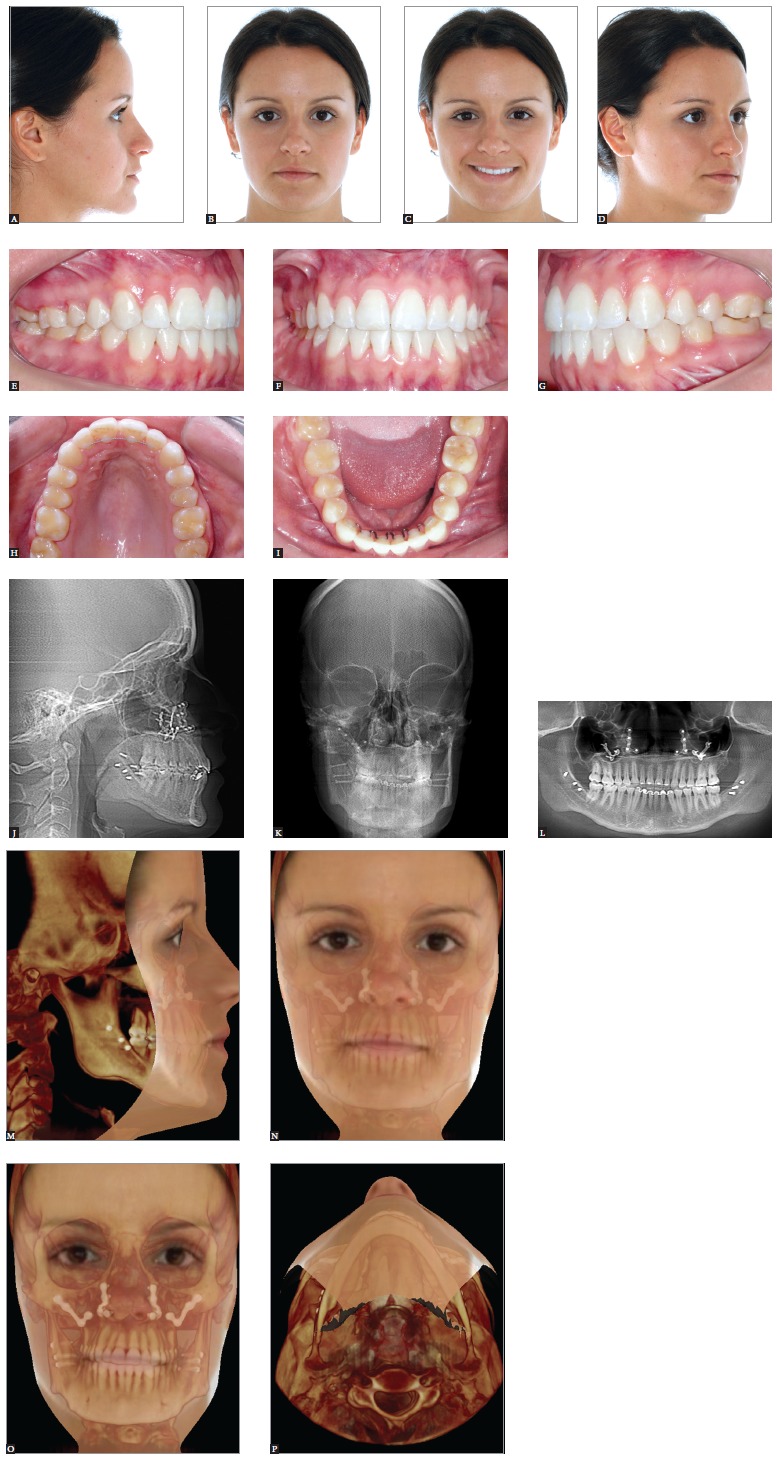
- Treatment outcomes for the patient presented in Figure 8. Final extraoral (A, B and C) and intraoral (D, E, F, G, H and I) photographs. Profile, posterior-anterior and panoramic radiographs (J, K and L).

Figure 10 (continuation) - CBCT scans with soft tissues overlapping hard tissues (M, N, O and P).

**Figure 11:**
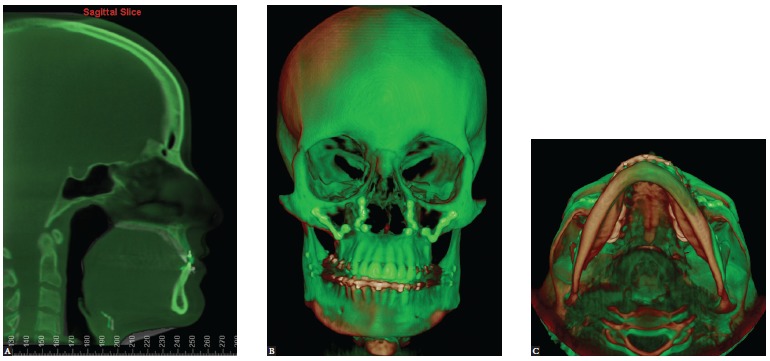
- Tomographic superimposition of patient presented in Figures 8 to 10 evincing changes before and after surgical correction of facial asymmetry (A, B and C). Surgical maxillary advancement of 4 mm was carried out, in addition to 1.5-mm impaction in the anterior region, 2-mm asymmetrical impaction in the posterior region on the right side and 2.5-mm asymmetrical impaction in the posterior region on the left side. The mandible was rotated for asymmetry correction

It is worth noting that accurate facial asymmetry correction is a major challenge, even when it is achieved by means of an orthodontic-surgical approach. This is because even though skeletal symmetry is achieved after the intervention, the asymmetrical growth of soft tissues occurring throughout the years is not usually corrected by surgery. Furthermore, some asymmetrical craniofacial regions oftentimes cannot be corrected by means of conventional surgical techniques. Thus, patients should be informed that in spite of successful correction of bone deviation, some asymmetrical contour might remain after orthognathic surgery.^12^^,^ 13 ^,^ 19 ^,^ 20

## FINAL CONSIDERATIONS

In spite of being highly prevalent in the overall population, facial asymmetry is scarcely addressed in dental literature. There is a lack of epidemiological studies, as well as histological and genetic research aiming at determining the real etiology and the factors associated with such disharmony. 

It significantly affects patients' smile and esthetics, and its correction is a major challenge posed to clinicians. Should it be of mild or severe magnitude, asymmetry needs intense orthodontic/orthopedic correction combined or not with orthognathic surgery.

Therefore, it should be highlighted that in-depth knowledge of facial asymmetry deserves special attention given by orthodontists who should be able to properly qualify all the features involved, in addition to quantifying the magnitude of disharmony, so as to provide patients with the best treatment possible. 
